# Traditional Chinese Medicine in Treating Influenza: From Basic Science to Clinical Applications

**DOI:** 10.3389/fphar.2020.575803

**Published:** 2020-09-15

**Authors:** Yibai Xiong, Na Xiao Li, Naifang Duan, Bin Liu, Hui Zhu, Chi Zhang, Li Li, Cheng Lu, Luqi Huang

**Affiliations:** ^1^ Institute of Basic Research in Clinical Medicine, China Academy of Chinese Medical Sciences, Beijing, China; ^2^ China Academy of Chinese Medical Sciences, Beijing, China; ^3^ Dongzhimen Hospital, Beijing University of Chinese Medicine, Beijing, China

**Keywords:** influenza, Traditional Chinese Medicine, herbal medicine, influenza symptoms, anti-influenza virus, review

## Abstract

Influenza infection is a highly contagious, acute febrile respiratory disease caused by the influenza virus. Traditional Chinese Medicine (TCM) has dominated plenty of theoretical and practical approaches in the treatment of influenza. It is, therefore, important to highlight the effects of TCM in the clinical treatment of influenza and their impact on inhibiting the growth of this virus in laboratory experiments. We scrutinized existing evidence on whether TCM is effective in clinical applications. Moreover, we described the potential mechanisms of TCM against the influenza virus. Our findings provide analytical evidence that supports the effectiveness of TCM in treating influenza infections as well as their mechanisms against this virus.

## Introduction

Influenza is an acute viral respiratory infection that was first described in the 4th century BC. At the time, it was estimated to have caused nearly 60,000 mortalities in Venice, Italy. Due to the impact of this disease in Venice, it was understood by folklore to be a punishment from God and was therefore named “Influenza (Devil)” (JJ, 2010). The influenza virus belongs to the Orthomyxoviridae family. It is a single-stranded negative-sense RNA virus exhibiting an eight-segmented genetic material (Brody, 2019). Its genetic material is made up of Polymerase Basic Protein 1 (PB1), Polymerase Basic Protein 2 (PB2), Polymerase Acidic (PA), Hemagglutinin (HA), Nucleoprotein (NP), Neuraminidase (NA), Matrix Protein (M), and Nonstructural Protein (NS) (Lu, 2008). Depending on the composition of its NP and M, this virus is grouped into three types (A, B, and C). Its subtypes are determined by the antigenicity of two surface glycoproteins, the 18 HA and 11 NA (Webster et al., 1992; Kawaoka, 2006; Huang et al., 2008). It is extremely susceptible to mutations because its RNA genome lacks proof-reading ability during replication as is the case with DNA genomes. Moreover, the mutations are also attributed to antigenic drifts. These mutating types propagate this virus, leading to epidemics in certain seasons (Cox and Subbarao, 2000; He et al., 2011; Kamali and Holodniy, 2013) and, notably, they hinder the development of new antiviral drugs. First-line antiviral drugs in the treatment of influenza rely on blocking the action of NP (Gaitonde et al., 2019). However, the side-effects of anti-NA drugs render many patients unavailable to treatment. In China, the broadly prescribed anti-influenza drugs include oseltamivir, zanamivir, and peramivir (Lee et al., 2017). Notably, Oseltamivir has been associated with resistance (Govorkova et al., 2001; Kiso et al., 2004; Moscona, 2004) and leads to immunocompromised conditions (Govorkova et al., 2001; Moscona, 2009; Panning, 2013). Zanamivir is administered through inhalation, and challenges over its use have been described (Shetty and Peek, 2012). Due to its limited bioavailability, peramivir can only be administered intravenously (Birnkrant and Cox, 2009). Therefore, these challenges call for more effort and attention toward developing fresh strategies to combat influenza. Previously, TCM demonstrated a positive anti-viral effect on influenza virus and alleviated the symptoms among patients (He et al., 2019; Nagai et al., 2019). In TCM theory, influenza disease was classified as “YI Disease” and was first reported in Huang Di Nei Jing. During the Han Dynasty, a theory of Shang Han (Treatise of Exogenous Febrile Diseases or Discourse on Cold-Damage Disorders) was written by Zhang Zhongjing to treat influenza. Later, during the Ming and Qing Dynasties, the emergence of “Wen Yi Lun (Analysis of Epidemic Warm Diseases)” revealed that TCM had contributed enormously to the prevention and treatment of influenza for thousands of years. Despite studies showing that TCMs are effective against influenza (Moscona, 2004; Tian et al., 2011), further studies on the development of novel anti-influenza agents are essential (Rajasekaran et al., 2013; Chang et al., 2014; Ding et al., 2014; Li C. et al., 2015). Herein, we summarize the impacts of TCM in treating influenza from basic experiments to clinical applications.

## Confirmed in Clinical Application

In Asia and some countries in Europe, TCM has been proven to be effective and secure for treating influenza (Moscona, 2004). Traditional Chinese Medicine (TCM) subdivides influenza into cold syndrome and heat syndrome. The major symptoms of wind-cold type include severe cold, light heat, lack of sweating, headache, sore limbs, and stuffy nose. On the other hand, high fever, a mild cold, headache, sore throat, cough are the primary symptoms of wind-heat type. By relying on the concept of evidence-based medicine, several clinical trials have been conducted to verify the efficacy and safety of TCM.

### Meta-Analysis

A meta-analysis enrolled 30 studies consisting of 3,444 cases in all to investigate efficacy and safety of TCM in the treatment of influenza infection. Our findings indicated that the mean time fever resolution of the TCM treatment group was statistically significant compared to the control group. Additionally, the synergistic effects of TCM and conventional medicines on viral infections were better compared to the control group (Li J. H. et al., 2016). Further, a different TCM prescription from Shang Han Lun, ma-huang-tang(5 g of *ephedra equisetina bunge*, 5 g of *prunus armeniaca L.*, 4 g of *cinnamomum verum J.Presl*, and 1.5 g of *glycyrrhiza uralensis fisch. ex DC.*) was confirmed that it alleviated fever when singly administered or in combination with neuraminidase inhibitors (NAIs) and might be a well-tolerated treatment as reported by a systematic review and meta-analysis conducted by Japanese scientists (Yoshino et al., 2019). Andrographis paniculata was indicated to be beneficial and safe for relieving influenza symptoms and shortening time to symptom resolution (Hu et al., 2017).Conclusively, TCM are validated in lessening influenza symptoms, such as duration for defervescence and cough.

### Randomized Clinical Trial (RCT)

Researchers conducted a randomized trial for comparing the efficacy and safety of oseltamivir and maxingshigan-yinqiaosan [6 g of *honey-fried ephedra equisetina bunge*; 10 g of *anemarrhena asphodeloides bunge*; 15 g of *artemisia annua L.*; 30 g of *gypsum fibrosum*; 15 g of *lonicera japonica thunb.*; 15 g of *scutellaria baicalensis georgi*; 15 g of *stir-baked prunus armeniaca L*.; 15 g of *forsythia suspensa (thunb.) vahl*; 6 g of *mentha canadensis L.*; 15 g of *fritillaria thunbergii miq.*; 15 g of *arctium lappa L*.; and 10 g of *glycyrrhiza uralensis fisch. ex DC.*] originating from Shang Han Lun and Wen Bing Tiao Bian, in treating uncomplicated influenza. Results have shown that singular use or combinations of oseltamivir and maxingshigan-yinqiaosan minimized defervescence time in patients diagnosed with influenza viral infections (Wang et al., 2011). It was, therefore, proposed that maxingshigan-yinqiaosan in combination with oseltamivir be utilized as an alternative medication in the treatment of influenza viral infections. In a multi-centric, randomized, double-blind, and placebo-controlled trial, a total of 480 adults with influenza symptoms were recruited. It was observed that TCM increased recovery by 17% (p < 0.001) and lowered disease severity (evaluated through the median symptom score) by 50% (p < 0.001) (Wang et al., 2010). Moreover, 136 influenza patients with wind-heat affecting Fei syndrome (WHAFS) were prescribed with Jinhua Qinggan Granules (JHG) that have been developed by experts in recent years. This preparation has been based on findings in a double-blind randomized control trial. In this trial, the duration of defervescence, the defervescence rate, the efficacy of TCM, the alleviation rate of primary symptoms and physical signs of influenza, the absence of viral nucleic acid in pharyngeal secretions, and safety indices were assessed. It was shown that JHG was efficient and safe in treating patients with WHAFS (Li et al., 2013). Japanese doctors have conventionally prescribed TCM to treat influenza. This is based on findings of a randomized controlled trial that compared the efficacy of TCMs with NP inhibitors in treating influenza. It has been established that TCM was effectively tolerated when used to treat influenza. Meanwhile, TCM and NP inhibitors exhibited equivalent clinical efficacies against the influenza virus (Nabeshima et al., 2012). In the US, a randomized, double-blind placebo-controlled study established that *Echinacea angustifolia* DC, a compound used in herbal tea preparations was potent in alleviating influenza symptoms such as stuffiness, scratchy throat and fever (Lindenmuth and Lindenmuth, 2000). In agreement with our meta-analysis, RCTs demonstrated that TCM abbreviated fever time and showed a satisfactory impact in relieving cough and sore throat. In **Table 1**, we summarized the efficacy of TCMs in treating influenza (**Table 1**).

**Table 1:** Distribution of influenza article types and outcome indicators.

Study Design	Source	Groups	N	Outcomes							
				Main Outcomes						Secondary Outcomes	
				Fever	Headache	Myalgia	Cough	Sorethroat	Malaise	Hospitalization	Side effects
**Meta**	Li J. H. et al., 2016	1.Ma Huang Tang plus NAIs vs. NAIs 2. Ma Huang Tang vs. NAIs	30 studies, 3,444 cases	↑	↑	↑			↑	↑	↑
	Yoshino et al., 2019 Hu et al., 2017	1.TCM VS oseltamivir 2.TCM + oseltamivir vs. oseltamivir Andrographis paniculata vs. placebo	12 studies, 1,170 cases 33 studies, 7,175 cases	↑			↑	↑		↑	
**Randomized Controlled Trial**	Wang et al., 2011	maxingshigan–yinqiaosan vs. Oseltamivir	410 cases	↑	↑		↑	↑			
	Wang et al., 2010	Antiwei vs. placebo	480 cases	↑	↑	↑	↑	↑			
	Li et al., 2013	Echinacea vs. Placebo	136 cases								
	Nabeshima et al., 2012	Jinhua Qinggan Granules vs. Placebo	28 cases	↑	↑	↑	↑	↑			↑
	Lindenmuth et al., 2013	Maoto VS oseltamivir vs. zanamivir	95 cases	↑	↑	↑	↑	↑			↑

The upward arrow represents the relief of symptoms after treatment.

### Retrospective Analysis

Besides meta-analysis RCT, researchers analyzed the duration of viral shedding in influenza patients admitted and administered with TCM in China. A total of 963 patients diagnosed with influenzavirus infection between May and July 2009 were recruited. The study showed that TCM therapy contributed to viral shedding among patients with a temperature of ≥ 38°C (Wang et al., 2012). Overall, these studies demonstrated that, besides alleviating symptoms of influenza patients, such as fever and cough, TCM have been proved to contribute to viral shedding.

### Use of TCM in Treating Influenza Among Children and During Pregnancy

Treatment of influenza among children and pregnant women is challenging. However, other than effectively alleviating symptoms, TCM can compensate for deficiencies that cannot be bridged by conventional medicines in pregnant women. Children is the major group affected by influenza virus as they are usually vulnerable to complications. Moreover, the response of children to drug-drug metabolism is profoundly unique from that of adults. Therefore, it is essential to analyze the effects of TCM on children. It is, therefore, essential to analyze the effects of TCM on children. As mentioned above, ma-huang-tang was expansively used in China to manage children diagnosed with influenza. Ma-huang-tang has been widely used in China to manage influenza diagnoses among children. Without exhibiting adverse effects, it has been validated to be clinically useful in neonates, infants, and children with febrile viral symptoms (Nishimura et al., 2009). Additionally, it has been shown that it might potentially be useful in patients aged ≤5 who have a low sensitivity to oseltamivir and experience problems when using zanamivir (Toriumi et al., 2012). Comparison of the efficacies of ma-huang-tang and oseltamivir was evaluated among children with type A influenza. Results showed that ma-huang-tang in combination with oseltamivir was capable of abbreviating fever duration when compared to the group administered with oseltamivir only (Kubo and Nishimura, 2007). Elsewhere, a systematic review focusing on the effects of *Andrographis paniculata* *(burm.f.) nees* on influenza infections among children reported that this herb minimized the duration of cough, sore throat and morbidity time compared to conventional medicines (Hu et al., 2017). The use of TCM aerosols in treating infantile influenza infections showed an effective rate of 99.03% with a cure rate of 65.38% (Ma et al., 2000). An investigation on the clinical efficacy of a sachet of Chinese herbs in preventing influenza among 239 children from Shanghai Baoshan Xubeihong Art Kindergarten revealed that the sachet alleviated symptoms such as fever, rhinocleisis, runny nose, and throat congestions. Equally, the incidence rates of influenza in the treatment group were low compared to the control group (Liu et al., 2010). Reports indicate that TCM is a valuable option during pregnancy. For instance, when influenza outbreaks occurred in the Middle East, the prevalence in the use of TCM varied between 22.3%–82.3% in pregnant women (John and Shantakumari, 2015). Also, Across-sectional study conducted in the Central Appalachian Region showed that *Trigonella foenum-graecum* *L.* was regularly used in treating Influenza (Alwhaibi et al., 2017). More and more RCTs are focusing on the efficacy and safety of combination use of TCM and conventional therapy (He et al., 2019). In summary, there is potential value in using TCM to treat influenza among children and pregnant women; nevertheless, more investigations are necessary for clinical validations.

## Basic Experiments

### Direct Anti-Influenza Virus by Interfering the Invasion Process

In the clinical set-up, we have witnessed the effects of TCM in improving the symptoms of influenza and cutting the course of the disease. However, how TCM act on influenza virus and its effects on the host requires a comprehensive analysis using basic experiments. Recently, researchers have constantly been drawn to investigate the therapeutic effects of TCM. Several types of TCM including, *coptis deltoidea* (*C.Y.Cheng & P.K.Hsiao)*, *Isatis tinctoria* *L.*, *Lonicera japonica* *thunb.*, *scutellaria baicalensis* georgi., *cyrtomium fortunei* J.Sm., *Houttuynia cordata* *thunb.*, *gardenia jasminoides* *J.Ellis*, and *chrysanthemum indicum* *L*., and other TCM prescriptions have been proven to effectively suppress influenza virus (Han et al., 2016).

#### Single Herbal Medicine

**Figure 1:**
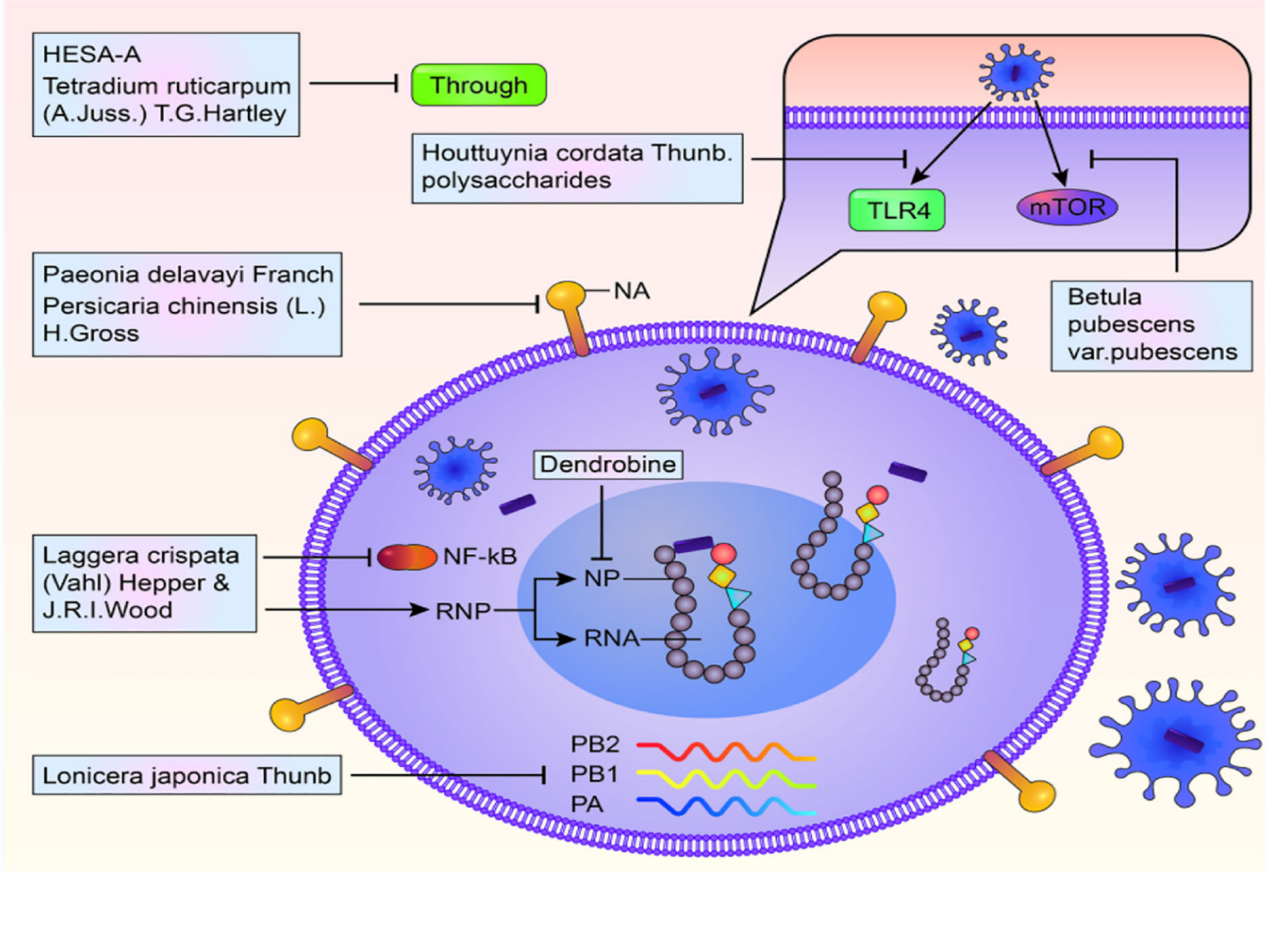
Multiple targets and multiple pathways to inhibit influenza virus.

The influenza virus invades the human body in six steps, that is, adsorption, penetration, shelling, biosynthesis, assembly, and release. As its mode of action, TCM targets these six steps. i) The extracts from single herbal medicine: *Lonicera japonica thunb.* HS-encoded atypical microRNA was verified to inhibit influenza viral replication *in vitro* and *in vivo*. Furthermore, HS decoction significantly minimized mice mortalities from influenza virus infection (Zhou et al., 2015). Extracts of *Laggera crispata (vahl) (Hepper & J.R.I.Wood*), a TCM have been shown to inhibit NA activity. It has also been reported that this compound could suppress NF-κB signaling pathways and viral RNP complexes from the nucleus thereby inhibiting viral replication (Guan et al., 2017). Besides blocking viral replication, TCM enhances the prognosis of mice infected by influenza. Yan et al. evaluated the effects of berberine, a natural isoquinoline alkaloid isolated from *coptis chinensis* *franch.* on influenza virus. Their results showed that berberine suppressed viral replication in A549 cells and the lungs of mice. Compared to conventional medicines, this compound exhibited significant anti-inflammatory effects on the pulmonary system and reduced necrosis in mice. It was also observed that inflammatory cell infiltrations and pulmonary edemas due to viral infections in mice were reduced (Yan Y. Q. et al., 2018). Houttuynia cordata Thunb (HCP), a TCM, plays a pivotal role in the treatment of bacterial and viral respiratory infections. Accompanied by a reduction in virus replication, HCP is reported to increase the overall survival rate of mice infected with influenza. Further studies have proved that it might be associated with the decrease in the concentration of pulmonary proinflammatory cytokines/chemokines and the number of intestinal goblet cells, which resulting in upregulation of sIgA and tight junction protein (ZO-1) in the intestine (Zhu et al., 2018). Researchers at the Kitasato Institute in Japan extracted flavonoids from the roots and leaves of *Astragalus mongholicus* *bunge* and confirmed their capacity in preventing the growth of influenza virus. It has been reported that glycyrrhizic acid significantly inhibits influenza virus (Pompei et al., 1983; Baltina et al., 2009). Besides, extracts of *Paeonia delavayi* Franch. and *pogostemon cablin (blanco) benth.* were found to efficiently inhibit NP (Li J. et al., 2016; Liu et al., 2016). The roots of *Bupleurum marginatum* var. *stenophyllum* (*H.Wolff*) had inhibitory effects on the replication of influenza (Fang et al., 2017). Importantly, it has been confirmed that *Tetradium ruticarpum* (*A.Juss.*) *T.G.Hartley* effectively hindered viral attachment and penetration into host cells (Lin et al., 2016). ii) Single components: A study by Li et al. addressed that, Dendrobine, extracted from a traditional Chinese herb (*dendrobium nobile lindl.*), interfered with early viral replication and bound to the viral NP thereby restricting nuclear export of viral NP and its oligomerization (Li et al., 2017). HESA-A is a natural ingredient retrieved from marine herbs. This compound blocks viral penetration into the cell (Mehrbod et al., 2014). *Persicaria chinensis* *(l.) h. gross*, from Vietnam, was shown to target NP, including oseltamivir-resistant ones (Tran et al., 2017). These results show that single herbal medicines inhibit viral proliferation by targeting multiple sites and pathways during replication. These results are presented in **Figure 1**.

#### Traditional Chinese Herbal Prescription

Besides the single Chinese herbal medicine, traditional Chinese herbal prescription has precariously shaped the academic debate on influenza disease management. Huang-Lian-Jie-Du-Tang (composed of *coptis chinensis Franch.*, *scutellaria baicalensis georgi*, *phellodendron chinense C.K.Schneid.*, and *gardenia jasminoides J.Ellis* at the weight ratio of 3:2:2:3) (HLJDT) is a classical prescription composed of 4 TCM herbs that have been used to treat diverse diseases including influenza. In total, 13 compounds have been isolated from the plasma profile of HLJDT. These compounds include, jatrorrhizine, palmatine, epiberberine, geniposide, oroxylin A, berberine, coptisine, baicalein, wogonoside, phellodendrine, wogonin, oroxylin A-7-O-glucuronide, and baicalin. They have been shown to be potent NA-1 inhibitors (Zhou et al., 2017). Xin-Jia-Xiang-Ru-Yin [(composed of *mosla chinensis* maxim., *lonicera japonica thunb.*, *dolichos lablab* L., *magnolia officinalis rehd. et wils*. and *forsythia suspensa (thunb.) vahl* at the weight ratio of 2:3:3:2:3)], a traditional Chinese prescription used for managing summer influenza in China, has been demonstrated to integrate antiviral therapy and immune modulation effects by lowering the expression of Interferon-γ (IFN-γ) and Signal transducer and activator of transcription-1 (STAT1), resulting to reduced inflammation hence harboring key therapeutic benefits against summer influenza (Li et al., 2018). The Re Du Ping intravenous injection, a TCM can adsorb the virus into host cells and inhibit their proliferation. This injection also has a direct viricidal effect (Jing et al., 2010). Studies into the pharmacodynamics and molecular mechanisms of Qingqi Liangying granules have reported that they participate in Wnt signaling pathways by regulating virus invasions on epithelial cell signaling pathway, thereby, inhibiting viral replication (Wang et al., 2017). Chinese classical herbal prescription was also used in the prevention of influenza. It was reported to reduce the vulnerability of cells to the invasion of influenza virus and alleviate viral induced lung lesions (Liu et al., 2013).

### In-Direct Anti-Influenza Virus by Regulating Host Immune System

In addition to the direct effect on the influenza virus, TCM regulates immune activation and signaling pathways that impact on the influenza virus. The host innate immune response to influenza virus occurs as follows: Infection with influenza virus stimulates the body’s innate immune response, which involves multiple effector cells, immune molecules, and factors, such as Interleukin-6 (IL-6) and Interferon-α (IFN-α), to effectively regulate viral replication (Hayden et al., 1998). Several experiments have confirmed that TCM is the most potent anti-inflammatory natural medicine which can modulate all kinds of cytokines and immune molecules.

#### Traditional Chinese Herbal Prescription

A study conducted on the mechanisms of TCM prescription in the treatment of influenza deduced that anti-inflammatory effects conferred by TCM protect against inflammatory injuries induced by the influenza virus in mice. These herbal medicines significantly reduce the expression of pro-inflammatory cytokines Tumor Necrosis Factor-α (TNF-α) and IL-6 in lung tissues of infected mice (Wang et al., 2005; Xu et al., 2014). Sheng Jiang San (SJS), (*rheum officinale* baill., *bombyx batryticatus*, *cicadae periostracum*, and *curcuma longa* *L*. in a ratio of 4:2:1:3), a traditional multi-herb prescription, used to treat influenza infections. Research has shown that SJS down-regulates TNF-α and up-regulates IL-2 in influenza infected mice. Equally, lung indices used to evaluate the level of inflammation and viral loads in SJS treated mice were markedly decreased compared to the controls (Zhang et al., 2018). Currently, Yinhuapinggan granule (YHPG), a Chinese drug with patent that originated from ma-huang-tang is used in the treatment of influenza. A study conducted by Du revealed that YHPG markedly inhibited replication of the influenza virus and significantly up-regulated the levels of IFN-β and IFN-stimulated genes (Mx-1, ISG-15, and ISG-56) compared to the control group (Du et al., 2018). The Yinqiao powder [15 g of *forsythia suspensa (thunb.) vahl*, 15 g of *lonicera confusa DC.*, 9 g of *platycodon grandiflorus (jacq.) A.DC.*, 9 g of *mentha canadensis L*., 6 g of *lophatherum gracile brongn.*, 5 g of *glycyrrhiza uralensis fisch. ex DC*., 6 g of *nepeta tenuifolia benth*., 6 g of g*lycine max (L.) merr.*, 6 g of *arctium lappa L.*, 10 g of *phragmites australis subsp*. australis]; The Xinjiaxiangruyin concoction [6 g of *mosla chinensis maxim.*, 9 g of *lonicera confusa DC.*, 9 g of *lablab purpureus subsp. purpureus*, 6 g of *magnolia officinalis rehder & E.H.Wilson*, 6 g of *forsythia suspensa (thunb.) vahl*]; and the Guizhi-and-Mahuang decoction [9 g of *ephedra sinica stapf*, 6 g of *cinnamomum cassia (L.) J.Presl*, 9 g of *prunus armeniaca L*., 6 g of *glycyrrhiza uralensis fisch. ex DC*., 9 g of *paeonia lactiflora pall*., 9 g of *zingiber officinale roscoe*, 3 g of *ziziphus jujuba mill*.] are widely applied in clinical influenza treatment. These compounds reduce the expression levels of TLR7, MyD88, IRAK4, and NF-κB thereby by regulating the Toll-Like Receptor7/Nuclear Factor Kappa-B (TLR7/NF-κB) signaling pathway (Fu et al., 2018). Yi-Zhi-Hao pellet (CYZH) is a popular Chinese prescription used in influenza treatment. Mechanistically, CYZH lacked an inhibitory effect on viral protein HA and IAV RNA-dependent RNA polymerase but inhibited IAV replication by activating the Nrf2/HO-1 pathway (Yin et al., 2017). Lianhuaqingwen, a TCM prescription alleviates viral-induced gene expressions of IL-6, IL-8, TNF-a, IP-10, and MCP-1 (Ding et al., 2017). Furthermore, Lianhuaqingwen treatment resulted in abnormal particle morphology of virion in cells (Runfeng et al., 2020). San Wu Huangqin (SWHD) decoction (*sophora flavescens*, *scutellaria baicalensis*, and *rehmannia glutinosa* at a ratio of 1:1:2) exhibits its anti-viral effects by regulating the immune system (Ma et al., 2018). The Ge Gen Decoction [12 g of *pueraria montana* var. lobata (Willd.) maesen & *S.M.Almeida ex sanjappa & predeep*, 9 g of *ephedra sinica* stapf, 6 g of *cinnamomum cassia* *(L.) J.Presl*, 6 g of *glycyrrhiza uralensis* fisch. ex *DC*., 6 g of *paeonia lactiflora* pall., 9 g of *zingiber officinale* roscoe, and 22 g of *ziziphus jujuba* *mill*.] decreased the expression of TNF-α and toll-like receptor 7 signaling pathways in influenza virus infected mice thereby reducing lung inflammation (Geng et al., 2019). Research endorsed that Gui Zhi Ma Huang Ge Ban Tang [9 g of *ephedra sinica* stapf, 6 g of *cinnamomum cassia* *(L.) J.Presl*, 9 g of *prunus amygdalus* *batsch*, and 6 g of *glycyrrhiza uralensis* *fisch. ex DC*., 9 g of *paeonia lactiflora* *pall*., 9 g of *zingiber officinale* *roscoe*, and 10 g of *ziziphus jujuba* *mill.*] exhibit a good ability to reduce the proportion of Helper T cell 1/Helper T cell 2(Th1/Th2) and Helper T cell 1/Regulatory cells (Th17/Treg) cells thus reducing lung inflammation (Qin et al., 2018). *Artemisia scoparia Waldst. & Kit.* is expansively distributed in Xinjiang China and reported to downregulate NF-κB signal pathway thereby inhibiting viral replication (Yan H. et al., 2018). Summarily, TCM regulates signaling pathways like NF-κB and inflammatory factors such as TNF-α and IL-6, that are important in modulating immune responses against the virus.

#### Single Herbal Medicine

Besides the TCM prescription, single herbal medicine indirectly plays a crucial role in anti-viral activity. i) The extracts from single herbal medicine: As mentioned above, *laggera pterodonta*, demonstrated a wide spectrum of anti-influenza virus activity *via* the NF-κB pathway, COX-2, and p38/MAPK pathway (Wang et al., 2017). Moreover, flavonoids extracted from *Scutellaria baicalensis* Georgi (FESR) could inhibit excessive activations of the complement system, thereby, improving acute lung injuries (Zhi et al., 2020). *Houttuynia cordata* thunb polysaccharide (HCP) aids systemic influenza treatment by locally acting on the intestines and balancing the microbiota (Chen et al., 2019). *Bupleurum falcatum* L., extracted from a traditional Chinese herb, was also found to be an immune modulator (Yao et al., 2018). *Isatis tinctoria* L., attenuates viral-induced NF-κB activation (Li et al., 2015). Research has shown that *Forsythia suspensa (thunb.) vahl* interferes with the budding process of newly formed virions and reduces influenza M1 protein that is necessary for viral spread (Law et al., 2017). ii) Single components: Baicalin, a popularly known herbal medicine ingredient in China for treating Fei-Re syndrome characterized by fever, and cough, can be extracted from *scutellaria baicalensis georgi*, which was reported to induce autophagy so as to inhibit mTOR signaling pathway. Thus, shedding more light on the development of novel anti-influenza drugs (Zhu et al., 2015). Quercetin-7-O-glucoside was discovered in many types of Chinese herb and demonstrated a strong inhibition against influenza A and B viruses by downsizing virus-induced ROS and autophagy formation (Gansukh et al., 2016). The polysaccharides extracted from the roots of *astragalus mongholicus Bunge* species, a famous TCM used for hundreds of years to improve QI with Chinese medicine theory, was ratified to modulate Th1/Th2 balance and secretions of IFN-γ, IL-17A, and IgG2a (Yakuboğulları et al., 2019). These studies testify that different types of TCMs possess remarkable potential in immunomodulation against influenza virus.

## Summary

In conclusion, TCM impacts on the prevention and treatment of influenza. It has a potential value in shorting fever durations and alleviating influenza symptoms among children and pregnant women. However, the side effects of TCM in children and pregnant women are still elusive, which needs more clinical trials about the safety and vivo toxicity. These medicines also regulate the immune system. Their modes of action involve inhibiting NA, viral replication, and stopping viral entry into the cell. The synergistic effects of TCM and conventional medicines are encouraging as an avenue for influenza therapy.

## Author Contributions 

YX: Conceptualization, Writing—original draft. ND: Conceptualization. XL: Writing—original draft. BL: Writing—original draft. HZ: Writing—original draft. CZ: Supervision. LL: Supervision. CL: Supervision. LH: Funding acquisition and supervision.

## Conflict of Interest

The authors declare that the research was conducted in the absence of any commercial or financial relationships that could be construed as a potential conflict of interest.

The handling editor declared a shared affiliation, though no other collaboration, with the authors at the time of the review.

## Abbreviations

TCM, Traditional Chinese Medicine; PB1, Polymerase Basic Protein 1; PB2, Polymerase Basic Protein 2; PA, Polymerase Acidic; HA, Hemagglutinin; NP, Nucleoprotein; M, Matrix Protein; NS, Nonstructural Protein; NA, Neuraminidase; NAIs, Neuraminidase Inhibitors; WHAFS, Wind-heat affecting Fei syndrome; RCT, Randomized Controlled Trial; HCP, Houttuynia cordata polysaccharide.
